# American Medical Association

**Published:** 1874-05-01

**Authors:** 


### AMERICAN MEDICAL ASSOCIATION.

ON the subject of "the reorganization" of the Association, the editor of the *Medical Record*, of New York, in the number for April 15th, has a page or more of editorial, which suggests the idea that our *confrere* must have been taking a Rip Van Winkle nap. He devotes a full column to the advocacy of a large Standing Committee on Ethics, which shall take the entire charge of all personal, ethical, or judicial questions, that the general business of the Association may be kept free from bickerings and useless discussions. He says: "At the last meeting in St. Louis, Dr. Davis made some propositions for the creation of this committee, but they were laid on the table." If our highly esteemed co-laborer of the *Record* will turn to pages 35 and 36 of the published Transactions for 1873, under the head of "Judicial Council," he will find fully adopted and incorporated into the Constitution of the Association the very provision which he advocates, under the impression that it was laid on the table.

The provision for a permanent Judicial Council, or large Ethical Committee, was not only fully provided for, at the last annual meeting, but the members were appointed, and the Council organized, as may be seen by referring to pages 55 and 111 of the Transactions. Dr. H. W. Dean, of Rochester, N. Y., is Chairman, and Dr. S. N. Benham, of Pittsburg, Pa., Secretary of the Council. The *Record* also refers to the propositions of Dr. Gross, made in 1872, for abolishing the committees on Medical Education, Medical Literature, etc., and substituting in their place addresses on Medicine, Surgery, etc., to be made in open session, by members selected for that purpose. Here, too, he may find the changes he advocates already made, by turning to the Transactions, pages 34–5–6. And if he will attend the meeting in Detroit during the first week in June, he may enjoy the great pleasure of listening to a public address on Surgery by Prof. Gross, himself.

The only important item of "reorganization" mentioned in the *Record*, which is not already adopted and in practical operation, is that in relation to the election of delegates; and for this, the necessary constitutional amendments were proposed, in due form, at the meeting in St. Louis, and are ready for adoption or rejection at the next meeting. Now, will the editor of the *Record* meet us in Detroit and give us his vote on their adoption? If so, we are sure he will not only get himself better posted concerning the doings of the Association, but he will find very much less to complain about than heretofore.

For proposed amendments, see Transactions, page 44.

